# Prehabilitation Program for Lung and Esophageal Cancers (Boosting Recovery and Activity Through Early Wellness): Protocol for a Nonrandomized Trial

**DOI:** 10.2196/60791

**Published:** 2025-03-10

**Authors:** Jodi E Langley, Daniel Sibley, Joy Chiekwe, Melanie R Keats, Stephanie Snow, Judith Purcell, Stephen Sollows, Leslie Hill, David Watton, Abbigael E Gaudry, Ibrahim Hashish, Alison Wallace

**Affiliations:** 1 Dalhousie University Halifax, NS Canada; 2 Beatrice Hunter Cancer Research Institute Halifax, NS Canada; 3 University of Toronto Toronto, ON Canada; 4 Mentor Primary Health Clinic Halifax, NS Canada; 5 YMCA of Nova Scotia Halifax, NS Canada; 6 Nova Scotia Health Halifax, NS Canada; 7 Nova Scotia Cancer Care Program Halifax, NS Canada; 8 Patient Partner Yarmouth, NS Canada

**Keywords:** cancer, prehabilitation, physical activity, lung, esophageal, wellness, surgical, candidacy, feasibility, implementation, community-based, coaching program, Canada, lung cancer, esophageal cancer, surgery, nonrandomized trial, mixed method

## Abstract

**Background:**

Cancer is the leading cause of death in Canada, responsible for 28.2% of all deaths. Based on surgical candidacy and disease status, both lung and esophageal cancer are treated through surgical resection by a thoracic surgeon. Although surgery contributes to improved outcomes, the 30-day postoperative mortality risks are as high as 10% and 2.8%, respectively. Evidence has shown that prehabilitation is a way in which patients can have improved postoperative outcomes. Prehabilitation is multimodal, often including some form of movement, nutrition, stress management, and smoking cessation. Given the complexity of the health care system, pragmatic trials are important methodological tools to assess internal validity and improve current practice under real-world conditions. Concurrently, using community resources is imperative to keep people active in their community and create sustainable programming.

**Objective:**

The Boosting Recovery and Activity Through Early Wellness (BREATHE WELL) study aims to explore the feasibility, implementation, and preliminary effectiveness of a clinically integrated, community-based, prehabilitation health coaching program. This includes nutrition, smoking cessation, sleep hygiene, and movement for individuals scheduled to undergo surgery for lung or esophageal cancer.

**Methods:**

This is a pilot, nonrandomized, pragmatic, repeated measures, mixed methods trial. We will recruit 32 participants diagnosed with lung or esophageal cancer and are scheduled to undergo surgical resection into the prehabilitation program, with 32 additional participants who decline participation to act as a control group. Participants who agree will then go through an 8-week tailored prehabilitation program (in person or virtual), covering movement, nutrition, stress management, nutrition, goal setting, and smoking cessation. They will complete 6 sessions prior to surgery and then have 4 sessions, 1×/week following surgery. Following the completion of the program, they will have 3 booster sessions via phone or Zoom (Zoom Video Communications). The primary outcome is feasibility: (1) recruitment feasibility—recruitment rate (the number of participants referred per month), enrollment rate (the number of enrolled participants divided by the number of referred participants), reasons for declining, and prehabilitation window (time between consent and surgery); and (2) intervention feasibility—adherence to the movement intervention, attrition, safety, study completion rate, and adverse events. Secondary outcomes include measures of preliminary effectiveness including patient-reported outcomes, such as well-being, fatigue, and functional measures. All measures will be assessed before, during, and after the prehabilitation program.

**Results:**

Enrollment has begun in January 2025, with 2 participants enrolled in the health coaching program. The full study is expected to be completed in approximately 3 years and be published in winter 2027.

**Conclusions:**

This study will inform the feasibility, implementation, and preliminary effectiveness of a clinically integrated, community-based, prehabilitation program in Nova Scotia, Canada, for people scheduled to undergo curative intent surgery for lung and esophageal cancer.

**Trial Registration:**

ClinicalTrials.gov NCT06354959; https://clinicaltrials.gov/study/NCT06354959

**International Registered Report Identifier (IRRID):**

PRR1-10.2196/60791

## Introduction

Cancer is the leading cause of death in Canada, responsible for 28.2% of all deaths [1]. Among these cancers, lung cancer is the most frequently diagnosed in the country, resulting in 25% of cancer-related deaths [2]. Esophageal cancer, while less common, presents unique challenges due to its effect on patients’ nutritional status, its tendency to metastasize rapidly, and, consequently, its poorer prognosis [3]. Based on surgical candidacy and disease stage, surgical resection by a thoracic surgeon is a mainstay of treatment for lung and esophageal cancers. Although surgery contributes to improved outcomes, the 30-day postoperative mortality risk are as high as 10% and 2.8% for lung and esophageal cancers, respectively [4,5]. Postoperative complications (eg, pneumonia and pain) pose a significant risk to patients undergoing curative-intent, lung and esophageal cancer surgeries [6]. These events not only increase the length of hospital stay but also negatively impact the patient experience, decrease quality of life, and result in substantial financial burdens for health care [7-10]. Therefore, it has been proposed that completing a prehabilitation program prior to undergoing surgery may lead to improved postoperative outcomes for patients. Prehabilitation refers to assessments and interventions initiated prior to treatment to create a physiological and psychosocial buffer against anticipated deconditioning, complications, and other comorbidities that typically occur as a result of treatment [11,12]. Prehabilitation is multimodal, often including some form of movement, nutrition, stress management, and smoking cessation.

Previous research has found that older adults (aged 70 years and older; American Society of Anesthesiologist score III/IV) who participated in prehabilitation experienced 50% less postoperative complications relative to the control group; this type of intervention was safe and feasible and showed cost-savings of up to €800 (~US $833.62) per patient [13]. Although prehabilitation interventions have generally shown benefit [14], there is still little uptake into the standard of care. This could be due to a wide variety of factors, including the complexity of implementing programs in a health care setting. Therefore, there needs to be more consideration for clinical care pathways, delivery strategies, and infrastructure and personnel for the local uptake of these programs.

Given the complexity of the health care system, pragmatic trials are important methodological tools to assess internal validity and improve current practice under real-world conditions [15]. Concurrently, using community resources is imperative to keep people active in their community and create sustainable programming. Practice-based evidence is an emerging field that strives to ensure research is applicable to real-world settings [16,17]. Practice-based evidence is derived from implementation science research methods, which assess effective intervention in real-world settings and provide insights into the system’s capacity and preparatory needs for dissemination and scalability [18]. The purpose of Boosting Recovery and Activity Through Early Wellness (BREATHE WELL) is to explore the feasibility, implementation, and preliminary effectiveness of a clinically integrated, community-based prehabilitation health coaching program, including nutrition, smoking cessation, sleep hygiene, and movement for individuals scheduled to undergo surgery for lung or esophageal cancer. Central to our research endeavor is the driving question: “How can a clinically integrated, community-based prehabilitation program be successfully implemented in the community, and can it lead to improved functional outcomes in patients undergoing surgery for lung or esophageal cancer?” This overarching question provides the study with its core focus and serves as a guiding force throughout the research process. The aim of this study is to investigate the implementation, feasibility, and effectiveness of BREATHE WELL, a community-based prehabilitation health coaching program specially designed for individuals scheduled to undergo surgery to address lung or esophageal cancer.

## Methods

### Design

**Figure 1:**
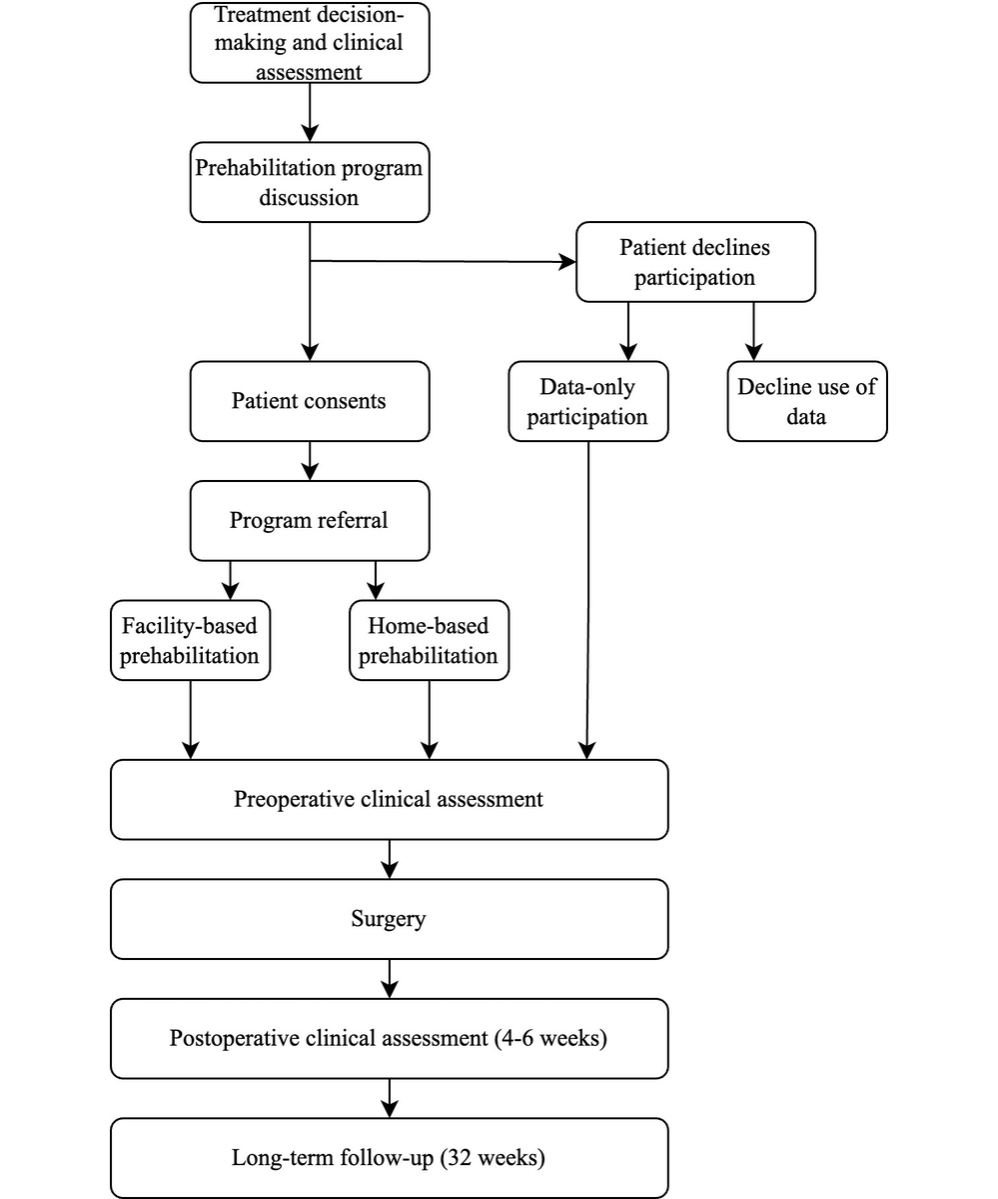
Participant flow.

This is a pilot, nonrandomized, pragmatic, repeated measures, mixed methods trial to assess the feasibility, implementation, and effectiveness of a clinically integrated, community-based, multimodal prehabilitation program for patients with lung and esophageal cancers in an urban academic health center in Halifax, Nova Scotia, Canada. Participant flow throughout the study is presented in Figure 1. This study aligns with the TREND (Transparent Reporting of Evaluations of Nonrandomized Design) checklist (Multimedia Appendix 1) for nonrandomized trials [19].

### Participants

Based on a monthly surgical volume of 10 patients per month to the referring surgeon, assuming an enrollment rate of 25% while accounting for 20% attrition within a 16-month recruitment window, we will seek to recruit 64 participants (aged 18 years and older) diagnosed with lung or esophageal cancer (32 in the program and 32 who decline). This is based on the number of surgical candidates that the current surgeon sees in a 2-year period. Participants will be identified based on the following inclusion criteria: (1) confirmed diagnosis of lung or esophageal cancer; (2) treatment plan includes surgery (at least 2 weeks from the time of consent) [20]; (3) fluent in English; and (4) surgical oncologist approval. Exclusion criteria include (1) unstable or symptomatic cardiac disease, musculoskeletal injury, or comorbid disease that precludes the ability to safely engage in physical activity or (2) significant cognitive limitations.

#### Enrollment and Assessment

Each potential participant will go through an assessment with the surgeon, including height, weight, blood pressure, heart rate, pulmonary function tests, grip strength, and a stair climb test as part of their clinical assessment. The thoracic surgeon will then raise the subject of prehabilitation with patients. At this point, patients will have the choice to participate in the prehabilitation program or not. If the participants do not agree to the study, they will continue with a standard of care. If a participant agrees to participate and provides written informed consent in accordance with the Nova Scotia Health Research Ethics Board, they will be referred to the research team and YMCA for immediate start of a prehabilitation program. Those who do not participate in prehabilitation will be used as a comparison group, through a waiver of consent; only those as part of the circle of care will pull these deidentified data for the research team.

#### Intervention

**Figure 2:**
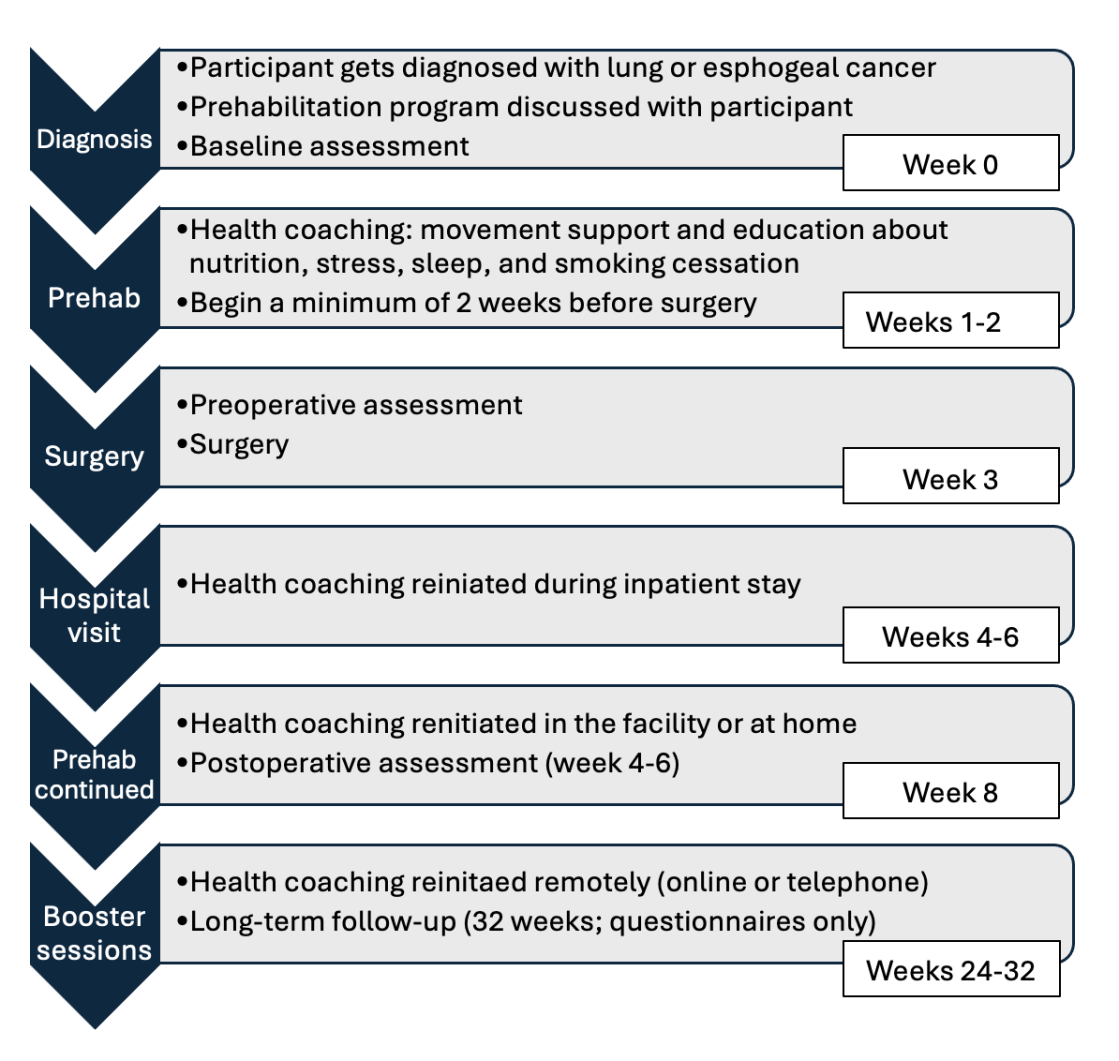
Timeline of prehabilitation intervention. Note that the “Prehab” phase may be longer than 1-2 weeks depending on the time from diagnosis to surgery. Prehab: prehabilitation.

Participants will first have an appointment with a community-based, qualified exercise professional to discuss a tailored prehabilitation program. This will be dependent on a current functional fitness assessment (completed by the surgeon), goals of care, and surgery date. Participants will receive a handbook that will guide them through this process. The handbook will include a calendar of events, a guide to prehabilitation modalities (eg, movement, nutrition, sleep hygiene, stress management, and smoking cessation), publicly available resources (eg, Canada’s Food Guide, CSEP 24-Hour Movement Guidelines, and Smoking Cessation guidelines), and helpful worksheets and other resources (Tobacco Free Nova Scotia Quit Line and mindfulness webinars). Participants will have the choice of delivery modality: either at home, virtual via Zoom (Zoom Video Communications), or at the nearest community-based YMCA. Both in-person and virtual programs will run in the same fashion. All participants will be given 2 resistance bands to assist with their at-home exercises. The intervention will last a total of approximately 8 weeks (Figure 2), depending on the time of surgery and postoperative follow-up. The intervention follows a tailored design, whereby the first 2-3 weeks provide more support, followed by weekly support and then biweekly support. This approach will help contribute to the autonomy of each patient. Following each counseling session, participants will engage in tailored movement sessions.

#### Health Coaching

Participants will work with a qualified exercise professional who is trained in motivational interviewing (MI) to promote positive lifestyle habits. Participants will meet virtually or in person for counseling and movement sessions. Participants will receive 6 health coaching sessions prior to surgery (approximately 3×/week), with 5 additional wellness sessions after surgery (1×/weekly in the first month), and a follow-up (approximately 4-6 weeks after surgery). Health coaching will last 10-15 minutes, and the aim of these MI-informed behavioral counseling sessions is to develop personalized goals. Through MI techniques, qualified exercise professionals will use a shared decision-making approach with participants to promote evidence-based lifestyle modification, while using publicly available, evidence-informed resources to educate the participant on healthy and positive lifestyle behaviors. Following health coaching, the coach will lead the participant through a 15-45-minute movement routine. The booster sessions will provide health coaching over the phone or via Zoom only (Table 1).

**Table 1:** Overview of health coaching sessions.

Session		Week		Topic		Goal of session
**Before surgery**						
	1	1	What to expect?		The goal is to have a clear understanding of what to expect from the diagnosis, surgery, and health coaching program. The qualified exercise professional will work with the participant to create a list of questions to discuss with their surgeon prior to their next visit. The qualified exercise professional will also start the process of what resources participants might need during their treatment, including Cancer Patient Pharmacare (which covers prescription cessation aids for example), Canadian Cancer Society patient supports (transportation supports), and other potential supportive services.	
	2	1	Understanding your body		The qualified exercise professional will go through how participants know their body the best, and things to look for when doing more movement. Key concepts of this session will be a rating of perceived exertion, use of our environment, and history of physical activity.	
	3	1	Movement + me		Participants will work with qualified exercise professionals to better understand what movement looks like for them and what type of movement they enjoy. Finding movement enjoyable is key to successful long-term movement.	
	4	2	Smoking cessation or sleep hygiene		If a participant reports current tobacco use, the qualified exercise professional will help the participant to understand the importance of smoking cessation as they prepare for surgery. This will include providing information about Tobacco Free NS and assisting with steps to access appropriate cessation resources. As well, participants will discuss sleep quality and the importance of sleep in preparation for and recovery from thoracic surgery.	
	5	2	Mindfulness		The qualified exercise professional will go through basic mindfulness attitudes, including patience, acceptance, and trust. The qualified exercise professional will review techniques for stress reduction and breathing.	
	6	2	Prepping for surgery		The qualified exercise professional will go through a reminder about things to bring with them to the hospital and talk about what to expect in the next few days. Participants will be reminded of the work they have done this far and be given a hospital date for their meet-up.	
**Surger**y						
	7	3	How are you feeling now?		The qualified exercise professional and participant will discuss how they are feeling and what supports the qualified exercise professional can offer to facilitate the transition from hospital to home setting.	
	8	4	Movement at home		The qualified exercise professional will assist the participant in better understanding movement at home and how they can make small movements as part of their everyday life. The focus of this session will be on doing small movement “snacks” and listening to their body.	
	9	5	Goal setting		Qualified exercise professionals will go through SMART^a^ goals with the participants. This will include long- and short-term goals with an understanding of upcoming treatment and how that will affect goals.	
	10	6	Nutrition		The qualified exercise professional will assist the participant in the understanding of food as a whole and how important nutrition is when undergoing treatments for cancer, this will include tips from Canada’s Food Guide. If needed, the qualified exercise professional will also have resources for the food bank and how to access these types of services in their community.	
	11	8	Long-term planning: what is next?		The qualified exercise professional will discuss a long-term plan for the participant to sustain healthy lifestyle habits. They will go through their movement notes together and find a movement plan that will work for this participant, whether that be at home, at a local gym, or a more structured program.	
**Follow-up appointment with surgeon**						
	12	16	Booster session: where we are now?		The qualified exercise professional will discuss how the participant is feeling now and revisit goals. If participants require further support, the qualified exercise professional will also do anything within their scope and also refer to the Community Health Team resources, which offer support on many wellness topics and are free to all Nova Scotia residents.	
	13	24	Booster session: where we are now?		The qualified exercise professional will discuss how the participant is feeling now and revisit goals.	
	14	32	Booster session: where we are now?		The qualified exercise professional will discuss how the participant is feeling now and revisit goals.	

^a^SMART: Specific, measurable, attainable, realistic, time orientated.

#### Movement Programming

All movement programming will be tailored to each individual participant’s current health status, goals, interests, and abilities. Each one-on-one movement session is expected to last 15-45 minutes and will include time for both warmup and cooldown. Participants will be instructed to begin at a light-to-moderate intensity (ie, 3-6 on a 10-point Borg Scale); systematically progress (ie, periodized); and combine aerobic and resistance training programs for 15-45 minutes, 3 days/week. Should a participant feel that any movement is beyond their comfort level or ability, they will be instructed to inform the qualified exercise professional so that the movement can be modified to better suit their needs (Table 2).

**Table 2:** Outline of a movement programming^a^.

	Description	Frequency	Intensity	Type	Time
Warmup	All sessions begin with a 5-minute warmup to gently increase heart rate and mobilize major muscle groups (RPE^b^ 1-3/10).	—^c^	—	—	—
Aerobic	—	3-7× per week (3 supervised and 0-4 unsupervised)	MICT^d^ (3-6 RPE) or HIIT^e^ (7-9/10 RPE)	Rhythmic repetitive movements (eg, walking, marching, cycling)	10-15 minutes
Resistance	—	3× per week	4-7/10 RPE	Targeting major muscle groups including: Legs (eg, sit to stand) Back (eg, horizontal row) Chest (eg, wall pushups) Core (eg, dead bug) Shoulders or arms (eg, shoulder press or bicep curl)	2 sets of 6-15 reps; 10-15 minutes
Balance	—	3-7× per week (3 supervised and 0-4 unsupervised)	4-7/10 RPE	Examples include single leg balance and tandem stance	2 × 30 seconds; 5 minutes
Flexibility	—	3-7× per week (3 supervised and 0-4 unsupervised)	4-7/10 RPE	Examples include hip flexor or quad stretch, hamstring stretch, and chest openers	2 × 20 seconds; 5 minutes
Locoregional	—	3-7× per week (3 supervised and 0-4 unsupervised)	4-7/10 RPE	Surgery-specific resistance and flexibility exercises (eg, swallowing exercises for esophageal participants)	2 × 20 seconds; 3-5 minutes
Cooldown	All sessions will conclude with a 5-minute cooldown to gently return heart rate to resting values (RPE 1-3/10).	—	—	—	—
Progression—aerobic	Sessions will begin at the lower limit of the desired range (eg, 3 sessions at 40% HRR^f^) and progress to the upper limit of the desired range (eg, 7 sessions at 70% HRR) as tolerated.	—	—	—	—
Progression—resistance, balance, and locoregional	Sessions will begin at the lower limit of the desired range (eg, 4/10 RPE) and progress to the upper limit of the desired range (eg, 7/10 RPE) by modifying repetitions, sets, or difficulty of exercises as tolerated.	—	—	—	—

^a^All programs will be tailored to each individual based on their functional level and environment.

^b^RPE: rating of perceived exertion.

^c^Not applicable.

^d^MICT: moderate intensity continuous training.

^e^HIIT; high intensity interval training.

^f^HRR: heart rate reserve.

#### Qualified Exercise Professional Training

Multiple YMCA-qualified exercise professionals will be trained in health coaching and MI to complete this study. This training will include (1) Thrive health services exercise oncology training [21], an exercise oncology-specific asynchronistic training module; (2) Health Coaching Certification [21], an evidence-based training that focuses on empowering health behavior change; and (3) completion of a full-day workshop on study protocol and MI techniques, including talks from experts in the field, individuals with lived experience of lung or esophageal cancer, and other health care professionals. Each counseling session will have a checklist and protocol for qualified exercise professionals to use outlining the targets and goals for each session. All qualified exercise professionals will be overseen by the YMCA clinical exercise physiologist who will offer advice and guidance on movement programming and health coaching sessions.

### Outcome Measures

Feasibility (primary outcome) will include (1) recruitment feasibility—recruitment rate (the number of participants referred per month), enrollment rate (the number of enrolled participants divided by the number of referred participants), reasons for declining, and prehabilitation window (time between consent and surgery); and (2) intervention feasibility (adherence to the movement intervention, attrition, safety, study completion rate, and adverse events). The feasibility target for recruitment is a 25% enrollment rate. The feasibility target for adherence is 70% or more, measured by attendance to movement sessions with the qualified exercise professional. The feasibility target for attrition is 20% or less [22]. A timeline of feasibility measures is available in Table 3.

**Table 3:** Timeline of program measures^a^.

		T0 (baseline)	T1 (before operation)	Surgery	T2 (after operation; weeks 4-6)		T3 (long-term follow-up; week 32)	
**Feasibility and acceptability**								
	Referral rate	✓						
	Referring provider enrollment	✓						
	Referred patient	✓						
	Enrollment rate	✓						
	Adherence		✓		✓		✓	
	Prehabilitation window		✓					
	Safety	✓	✓		✓		✓	
	Reasons for decline	✓						
	Attrition	✓	✓		✓		✓	
**Patient characteristics**								
	Demographic information	✓						
	Cancer (type and stage)	✓						
	Treatment Status	✓						
	Surgery type	✓						
**Resting, physical fitness, and patient-reported outcomes**								
	Height	✓	✓		✓			
	Weight	✓	✓		✓			
	Vitals	✓	✓		✓			
	Pulmonary function	✓	✓		✓			
	5TST^b^	✓	✓		✓			
	Stair-climb test	✓	✓		✓			
	Grip strength	✓	✓		✓			
	FACT-L^c^ or FACT-E^d^	✓	✓		✓		✓	
	FACIT-F^e^	✓	✓		✓		✓	
**Clinical outcomes**								
	Clavien-Dindo Surgical Complication (grade)			✓	✓			
	Postoperative hospital LOS^f^			✓	✓			
	Readmission and ED^g^ visits			✓	✓			
	Mortality			✓	✓			

^a^Pulmonary function test consists of tidal volume, forced vital capacity, vital capacity, and functional residual capacity. Vitals consist of blood pressure and pulse rate.

^b^5TSTS: 5 times sit to stand.

^c^FACT-L: Functional Assessment of Cancer Therapy—Lung.

^d^FACT-E: Functional Assessment of Cancer Therapy—Esophageal.

^e^FACIT-F: Functional Assessment of Chronic Illness Therapy—Fatigue.

^f^LOS: length of stay.

^g^ER: emergency department.

Participant outcome measures will include demographic information (age, sex, gender, socioeconomic status, race, marital status, educational level, and health knowledge), anthropometric measurements (height, weight, and BMI), medical information (surgery type, diagnosis, date of diagnosis, treatment status, and other chronic conditions), and patient-reported outcomes (PROs) that include quality of life (Functional Assessment of Cancer Therapy–Lung or Functional Assessment of Cancer Therapy–Esophageal) [23,24] and fatigue (Functional Assessment of Chronic Illness Therapy–Fatigue) [25]. Both the Functional Assessment of Cancer Therapy–Lung or Functional Assessment of Cancer Therapy–Esophageal and Functional Assessment of Chronic Illness Therapy–Fatigue have been extensively validated and are reliable and widely used tools in the oncology setting [23]. Participants will have the option of completing surveys using the web-based (REDCap [Research Electronic Data Capture]; Vanderbilt University) database or using a pen and paper–based survey. A comprehensive assessment of functionality will be conducted in person in the clinic by the surgeon. Functional assessments are based on the Canadian Society of Exercise Physiology’s Physical Activity Training for Health Protocol and include height, weight, resting heart rate and blood pressure, 5× chair sit-stand, stair climbing test, and grip strength. As part of standard of care, participants will undergo pulmonary function tests; these measures include tidal volume, forced vital capacity, vital capacity, and functional residual capacity.

The participants will be interviewed prior to the commencement of the program to better understand their perception of prehabilitation health coaching and to better understand their history with movement and healthy lifestyle behaviors. This will include questions that are based on past behaviors and future goals. This interview will be audio recorded and transcribed verbatim. A content analysis will be done using the Behavior Change Wheel and Theoretical Domains Frameworks [26,27]. This will aid in better understanding the behavior and barriers or facilitators to engage in the behavior. For sustained behavior change, participants need to have the Capability, Opportunity, and Motivation to perform the behavior [27]. Participants will also be interviewed at the end of the program to better understand their satisfaction with the program. This will also aid in understanding participants’ perceptions of where they are regarding long-term behavior change. Postprogram interviews will be conducted to better understand their perspective on the program and how or if they found the program beneficial; these interviews will probe the understanding of sustained health behavior change and program satisfaction.

Postoperative outcomes include length of hospital stay and postoperative complications. The severity of complications will be graded according to the Clavien-Dindo classification [28,29]. Return to the emergency department within 90 days after the operation and 90-day mortality will also be assessed.

### Statistical Analyses

The statistical analysis for this trial will be conducted with R (R Foundation for Statistical Computing). Participant characteristics will be summarized using descriptive statistics (mean, SD, frequency, and percentage). We will report on reasons for exclusion, attrition, and adverse events with frequency and percentages for each group. Safety will be determined by examining the total number of adverse events that occur over the duration of the program. Adherence to movement sessions will be summarized as a percentage (the number of movement sessions attended divided by the number of available movement sessions).

Baseline demographic and disease-related variables will be compared between prehabilitation and data-only participants using 1-way ANOVA for continuous variables and a chi-square test for categorical variables. The effectiveness analysis will include a linear mixed model to assess the difference in physical fitness and PROs from baseline (T0) to the preoperative (T1), postoperative (T2), 4-6 weeks postoperative (T3), and 32-week long-term follow-up (PROs only) time points, where the time point is the fixed effect and the individual participant is the random effect. A sensitivity analysis will be performed adjusting for sex, surgical type, and age. Linear mixed effect models will also be used to determine between-group differences. An α of .05 will be considered statistically significant. Point estimates and 95% CIs will be calculated for changes in the physical function and PROs at each timepoint and will also be used to provide important information for future sample size calculations. A Poisson regression will be used to estimate differences between groups for postoperative outcomes (ie, length of hospital stays, postoperative complications, return to emergency department, and mortality). Missing outcome data will be imputed using multiple imputation effects. Conclusions will be drawn using comparisons of each time point relative to the baseline.

Qualitative description is used to describe rather than interpret phenomena. Content analysis, the process of making sense of the meaning in the data will also be used during our thematic analysis. The research assistant will work with an experienced qualitative researcher to conduct multiple reviews of the transcripts and tapes (step 1) to familiarize themselves with the data. The analysis will be initiated as soon as the first interview is completed and continued concurrently with data collection. The analysis will examine what individual, structural, cultural, and institutional contexts are hypothesized to affect success. Additional codes will emerge to develop a thematic framework (step 2) that reflects the language and experiences of participants. An audit trial will be used to document the decision-making process. Sections of the transcripts will be charted into themes (step 3). Codes will be combined into themes during a series of research team meetings in which the relationships between behavior and the patient’s capability, opportunity, and motivation to do said behavior will be explored and summarized. Analysts will review the codes and associate themes multiple times to check for potential biases, to ensure they reflect participants’ words, and to improve and validate the interpretation (step 4) of the interviews. Additional interviews may be added when new themes emerge. Initial findings will be shared with a group of participants to help with interpretation and generate meaning from the data.

### Ethical Considerations

This prehabilitation study includes human participants and was ethically approved by the Nova Scotia Health Research Ethics Board (REB# 1030020) in April 2024. All participants will go through a written informed consent procedure and will be aware that this study is completely confidential and voluntary. Participants will be given a small amount of money to offset the price of gas and parking when participating in an in-person program.

### Author Reflexivity

This study brings together a multidisciplinary team with extensive expertise in research and clinical practice within the field of exercise oncology (JEL, DS, JC, and MRK); exercise measurement, evaluation, and prescription (JEL, DS, JC, and MRK); medical oncology (SS and AW); thoracic surgery (AW); policy implementation (LH and JP); and care experience (SS). The study team has a history of collaboration and has brought in key knowledge users to assist with the implementation of this project after the completion of research funding.

## Results

Enrollment has begun in January 2025, with 2 participants enrolled in the health coaching program. It is estimated that it will take approximately 24 months to recruit all participants and collect all measures; analyses will take approximately 6 months; and writing the final manuscript will take approximately 6 months to complete. The full study is expected to be completed in approximately 3 years and be published in winter 2027.

## Discussion

### Principal Findings

The current standard of care for patients undergoing surgery does not include prehabilitation, although multiple randomized controlled trials have shown the efficacy of these programs [9,10,20,30]. Evidence is growing for the use of prehabilitation in standard of care, yet its implementation in clinical settings is still limited. BREATHE WELL will inform clinicians and knowledge users how structured prehabilitation programming using community settings and resources can bridge the gap between health care and the community. To maximize care pathways, it is imperative that those suited for community programs are triaged accordingly. Further, it is important to ensure that community-based resources are well equipped and trained to work with patients in prehabilitation to allow for sustained health behavior change and safe movement programming. Individuals living with and beyond cancer during and after treatment benefit from engaging in movement; however, they often lack the knowledge, resources, and guidance on how to do so safely and effectively [31-35]. By using an “exercise and educate” model focused on MI and health coaching, the emphasis will be on empowering participants to see the long-term benefits of behavior change and increase overall well-being for individuals. Also, previous community-based physical activity interventions with individuals living with and beyond cancer do see a longer-term increase in physical activity [11]. This could be due to familiarity with the environment and having increased support upfront, which can lead to better physical activity adherence. This study builds on previous evidence that prehabilitation is efficacious, yet its uptake into the standard of care is still lacking [14].

As part of this study, the intentional engagement of knowledge users including key health system partners, clinical care programs, and patients as team members will ensure high-quality evidence. It also provides an excellent infrastructure for rapid dissemination of findings into policy, practice, and clinical care. Knowledge translation initiatives will be ongoing throughout this research, including integrated and end-of-grant knowledge translation. We will regularly engage with all research team members, which includes key knowledge users (JP and LH) and patient partners (SS). The preliminary findings will be shared, when necessary, at team meetings, and interim research summary reports will be circulated via email and existing webinars and continuing education infrastructure established through our team members and collaborators. To support the further development of this project, we will generate an end-of-study summary report of key findings to policy and clinical partners. Findings will be disseminated to lung and esophageal cancer–specific audiences in Canada and internationally through conferences (eg, Canadian Association of Thoracic Surgeons Annual Meeting, American Association of Thoracic Surgeons Annual Meeting, Canada Cancer Research Alliance Annual Meeting, Canadian Society for Exercise Physiology Annual Meeting, and American Society of Clinical Oncology) and will be published in open access journals (eg, *BMC Health Services Research*) to enhance accessibility and potential for global reach and impact. This research will also be disseminated to patients and lay audiences through infographics to increase accessibility to a wider nonacademic audience.

There are several strengths to this study: (1) a pragmatic design allows for a deeper understanding of implementation and feasibility, which will aid in further understanding of prehabilitation in real-world settings; (2) the use of community-based facilities allows for a strong likelihood of not only system-level sustainability but also greater confidence for individuals to adhere to physical activity upon completion of the prehabilitation program; and (3) the use of a standard-of-care comparator group allows for preliminary effect estimates to understand if prehabilitation improves quality of care. However, this study is not without limitations. First, we have limited our sample to patients who have undergone thoracic surgery, which may not be generalizable to other populations with cancer. Second, the prehabilitation window is short (2-3 weeks), which precludes physiological changes. This is due to constraints from the diagnosis to the surgery date; if participants do have a longer window, they will be offered a longer presurgical program. However, with continued support after the surgery, participants will be supported to create long-term positive lifestyle changes and habits. Finally, this is a nonrandomized trial, which may decrease internal validity. However, the focus of this study is pragmatic in nature and the primary outcome is feasibility.

### Conclusions

Prehabilitation is a key health intervention for those undergoing cancer surgery, with growing efficacy in the literature, yet the implementation of prehabilitation in real-world settings is lacking. BREATHE WELL will contribute to implementation science regarding approaches to support prehabilitation for surgical patients in a sustainable way that leverages the strengths of the health care and community setting.

## Appendix

Multimedia Appendix 1 TREND (Transparent Reporting of Evaluations of Nonrandomized Design) checklist.

## Abbreviations

BREATHE WELL: Boosting Recovery and Activity Through Early Wellness
MI: motivational interviewing
PRO: patient-reported outcome
REDCap: Research Electronic Data Capture
TREND: Transparent Reporting of Evaluations of Nonrandomized Design

## Data Availability

The original contributions presented in the study are included in the paper. Any further inquiries can be directed to the corresponding author. There are no data in the protocol paper.
